# The Survival of Roma Minority Patients on Chronic Hemodialysis Therapy - A Romanian Multicenter Survey

**DOI:** 10.1371/journal.pone.0155271

**Published:** 2016-05-19

**Authors:** Florica Gadalean, Daniel Lighezan, Dana Stoian, Oana Schiller, Romulus Timar, Bogdan Timar, Flaviu Bob, Mihaela Dora Donciu, Mircea Munteanu, Adelina Mihaescu, Adrian Covic, Adalbert Schiller

**Affiliations:** 1 Department of Nephrology, ‘Victor Babes’ University of Medicine and Pharmacy, County Emergency Hospital, Timisoara, Romania; 2 Department of Internal Medicine, ‘Victor Babes’ University of Medicine and Pharmacy, Municipal Clinical Emergency Hospital, Timisoara, Romania; 3 Department of Obstetrics and Gynecology, ‘Victor Babes’ University of Medicine and Pharmacy, Municipal Hospital, Timisoara, Romania; 4 B Braun Avitum Dialysis Center Timisoara, Timisoara, Romania; 5 Department of Diabetes and Metabolic Diseases, ‘Victor Babes’ University of Medicine and Pharmacy, County Emergency Hospital, Timisoara, Romania; 6 Department of Medical Informatics and Biostatistics, ‘Victor Babes’ University of Medicine and Pharmacy, County Emergency Hospital, Timisoara, Romania; 7 Department of Nephrology and Internal Medicine, University of Medicine “Gr. T. Popa” Iasi, Hospital “C. I. Parhon” Iasi, Iasi, Romania; Postgraduate Medical Institute, INDIA

## Abstract

**Objective:**

The Roma minority represents the largest ethnic group in Central and South-East European countries. Data regarding the mortality in Roma hemodialysis subjects are limited. We evaluated the 3 year mortality of ESRD Roma patients treated with hemodialysis (HD).

**Study Design and Setting:**

Our prospective cohort study included 600 ESRD patients on HD therapy recruited from 7 HD centers, from the main geographical regions of Romania. The median age of the patients was 56 (19) years, 332 (55.3%) being males, 51 (8.5%) having Roma ethnicity.

**Results:**

Roma ESRD patients initiate dialysis at a younger age, 47.8 years vs. 52.3 years (P = 0.017), present higher serum albumin (P = 0.013) and higher serum phosphate levels (P = 0.021). In the Roma group, the overall 3 year mortality was higher when compared to Caucasians (33.3% vs. 24.8%). The multivariate survival analysis revealed that being of Roma ethnicity is an independent risk factor for mortality (HR = 1.74; 95% CI = 1.04–2.91; P = 0.035).

**Conclusions:**

Roma patients with ESRD initiate HD therapy at a younger age as compared to Caucasians. They have a higher 3 year mortality rate and are dying at a younger age. Roma ethnicity represents an independent risk factor for mortality in our cohort.

## Introduction

Recent epidemiology data have shown that the Roma minority represents the largest ethnic group in Central and South-East European countries (up to7-9% of the population) [1].

Several studies showed a higher prevalence of chronic diseases (hypertension, diabetes, chronic respiratory disease, obesity, metabolic syndrome) in the Roma minority in comparison to the general population [2, 3, 4]. These findings, along with genetic, social, cultural and economic contributors, may explain the 20% prevalence of chronic kidney disease (CKD) in this ethnic group [4], with a relative risk of progressing to end stage renal disease (ESRD) 1.34 times higher comparing to the general population [5]. However, there is no data about the outcomes of the Roma minority with ESRD undergoing HD therapy. According to our knowledge this is the first multicenter study to address this issue.

## Subjects and Methods

In this prospective observational study we included all 600 ESRD patients under chronic HD therapy on 1^st^ of November 2010 (332 men and 268 women) in 7 Romanian centers. The cohort was followed-up until 31^st^ of December 2013, or until death. No patient was lost to follow-up.

At inclusion, patient’s data were obtained from their medical records. Dialysis vintage was defined as the time between the first day of dialysis treatment and the study entry date. A routine, complete hematologic and biochemical panel of analysis was performed according to the protocols of the Romanian Ministry of Health (assessment of anemia, inflammatory status, CKD-MBD, liver disease markers and hepatitis virus infection). Patients were treated with high flux, high surface, polysulfone (Xevonta) filters (not reused) and ready-to-use dialysis fluid (B. Braun acidic bicarbonate HD concentrate). Anemia and CKD-MBD were treated according to the KDIGO guidelines [6, 7].

Ethnicity was established based on patient’s self-identification and the appraisal of the nephrologist.

### Outcome analysis

Survival time was calculated from the study entry date (1^st^ November 2010) to the date of death or the administrative end of the study (31^st^ of December 2013). We assessed the association between ethnicity and all-cause mortality using Cox-proportional hazard models in which patients remained at risk until death or the end of the study. In the adjusted analysis of survival differences, the following covariates were included: ethnicity (Roma vs. Caucasian), patient’s age, HD duration, body mass index (BMI), hemoglobin, vitamin D levels, history of coronary artery disease, peripheral vascular disease, stroke, hepatitis virus infection and type 2 diabetes mellitus (DM).

### Statistical analysis

Data were collected and analyzed using the SPSS v.17 software suite (SPSS Inc. Chicago, IL, USA) and are presented as mean ± standard deviations for continuous variables with Gaussian distribution, median (interquartile range) for continuous variables without Gaussian distribution, or percentages for categorical variables. Survival was analyzed with Hazard Ratio (HR) method and presented using Kaplan-Meier diagrams.

To assess the significance of the differences between groups, the Student t-test (means, Gaussian populations), Mann-Whitney-U test (medians, non-Gaussian populations), Chi-square (proportions) and log-rank test (differences between survival curves and hazard ratio) were used. Continuous variable distributions were tested for normality using the Shapiro-Wilk test, and the Levene’s test for equality of variances. For evaluating the involvement of more confounding factors in time-related risk, Cox proportional-hazards models were constructed. The acceptance of a predictor in the equation was performed according to a backward Wald principle, having an entry probability threshold of 0.05 respectively removal probability threshold at 0.10. A p value of <0.05 was considered as the threshold for statistical significance.

The baseline characteristics of the cohort are presented in Table 1.

**Table 1:** The baseline characteristics of the studied cohort.

Studied parameter	Value
Roma (%)	51 (8.5%)
Men (%)	332 (55.3%)
Age—median (IQR)	56 (19) years
Time from first HD session–median (IQR)	2.7 (5) years
Weekly HD time–median (IQR)	12 (3) hours
eKt/V–mean ± SD	1.41 ± 0.31
Filtering surface–median (IQR)	1.5 (0.3) m^2^
Blood flow–median (IQR)	300 (40) ml/min
BMI–mean ± SD	25.5 ± 4.7 kg/m^2^
Coronary artery disease (%)	375 (62.5%)
Peripheral vascular disease (%)	150 (25.0%)
Stroke (%)	98 (16.3%)
Type 2 Diabetes Mellitus (%)	92 (15.3%)
Hepatitis B virus infection (%)	57 (9.5%)
Hepatitis C virus infection (%)	164 (27.3%)

BMI–body mass index

### Ethics statement

The study was approved by the Ethical Committee of BBraun Avitum Ltd Romania and the Ethical Committee for Human Research of the University of Medicine and Pharmacy Timisoara. Every patient provided written informed consent before enrollment. The study have been performed in accordance with the ethical standards as laid down in the 1964 Declaration of Helsinki and its later amendments.

## Results

The studied group was divided in two subgroups in respect to their ethnicity: Caucasian patients (n = 549) respectively Roma patients (n = 51) and compared accordingly. (Tables 2 and 3)

**Table 2:** Comparison of the studied parameters between Roma and Caucasian dialysis patients.

Parameter	Caucasian (n = 549)	Roma (n = 51)	p
Age (years)^a^	56 [19]	53 [19]	0.008 *
Dialysis duration (years)^a^	2.7 [5]	2.5 [5.5]	0.760
Age at the time of HD initiation (years)^a^	52.0 [19.85]	47.8 [16.6]	0.017
Weekly dialysis time (hours)^a^	12 [0]	12 [2]	0.755
eKtV^b^	1.40 ± 0.31	1.41 ± 0.31	0.859
Filtering surface (m^2^)^a^	1.8 [0.3]	1.5 [0.3]	0.187
Blood flow (mL/min)^a^	300 [40]	300 [5]	0.737
BMI (kg/m^2^) ^b^	25.4 ± 4.7	26.4 ± 6.6	0.275
Hemoglobin (g/dL) ^b^	11.2 ± 1.6	11.4 ± 1.3	0.356
Ferritin (ng/mL)^a^	633.4 [512.3]	624.7 [475.5]	0.199
TSAT (%) ^a^	18.7 [31.2]	18.7 [27.3]	0.726
hsCRP (mg/dL)^a^	1.8 [5.2]	1.5 [5.0]	0.780
Albumin (g/dL)^b^	4.3 ± 0.6	4.5 ± 0.6	0.013 *
Ca (mg/dL)^b^	8.5 ± 0.9	8.4 ± 0.9	0.116
PO4 (mg/dl)^b^	5.6 ± 1.7	6.3 ± 2.0	0.021 *
HCO3 (mEq/L)^b^	19.4 ± 4.3	20.2 ± 5.6	0.302
iPTH (pg/mL) ^a^	386.5 [655.0]	568.0 [439.1]	0.158
Cholecalciferol (ng/mL)^a^	19.9 [21.6]	20.9 [15.5]	0.294
ALP (UI/L) ^a^	95.0 [63.0]	115.5 [67.8]	0.027 *

*Differences are significant

^a^ Distributions are not Gaussian. Data is presented as median and [interquartile range]

^b^ Data is presented as mean±SD

BMI–body mass index, ALP—alkaline phosphatase, TSAT- transferrin saturation, hsCRP-high sensitive C-reactive protein, iPTH—intact parathyroid hormone

**Table 3:** The prevalence of co-morbidities in dialysis patients stratified by ethnicity.

Parameter	Caucasian (n = 549)	Roma (n = 51)	p
Coronary artery disease	342 (62.3%)	33 (64.7%)	0.734
Peripheral vascular disease	139 (25.4%)	11 (21.6%)	0.549
History of stroke	94 (17.11%)	4 (7.8%)	0.057
Type 2 Diabetes Mellitus	85 (15.5%)	7 (13.7%)	0.739
Hepatitis B virus infection	50 (9.1%)	7 (13.7%)	0.284
Hepatitis C virus infection	152 (27.7%)	12 (23.5%)	0.519

Data are presented as number of cases and (percentage from the total of the subgroup)

The Roma group had a significantly lower median age (53 years vs. 56 years, P = 0.008) having at the same time a similar duration of chronic HD (2.5 years vs. 2.7 years, P = 0.76). Also, Roma patients initiated maintenance HD at a significantly younger age (median age 47.8 [16.6] years vs. 52.0 [19.85] years, P = 0.017), leading to the conclusion that Roma ethnicity is associated with an earlier onset of end-stage renal disease.

Compared to the control group, in the Roma ethnicity group there were significantly higher mean PO4 levels (6.3 vs. 5.6 mg/dL, P = 0.021), alkaline phosphatase (115.5 vs. 95.0 U/L, P = 0.027) and serum albumin (4.5 vs. 4.3 mg/dL, P = 0.013). There were no other significant differences between the two groups. (Table 2).

Although the Roma ethnicity group included significantly younger patients than those included in the control group, there were no differences regarding the prevalence of comorbidities between the two groups. Treatment prescriptions with iron, erythropoietin, vitamin D, vitamin D receptor activator, and phosphate binders did not significantly differ in the two groups (data not shown).

During the 3 years of follow-up, 153 deaths were recorded, representing 25.5% of the enrolled patients. In the Roma group the overall mortality was higher compared to Caucasians (33.3% vs. 24.8%). The difference in the median of ages between the two groups, (the Roma group being younger by 3 years), suggests that Roma dialysis patients have a poorer outcome during dialysis therapy and are dying at a younger age compared to Caucasians.

**Fig 1:**
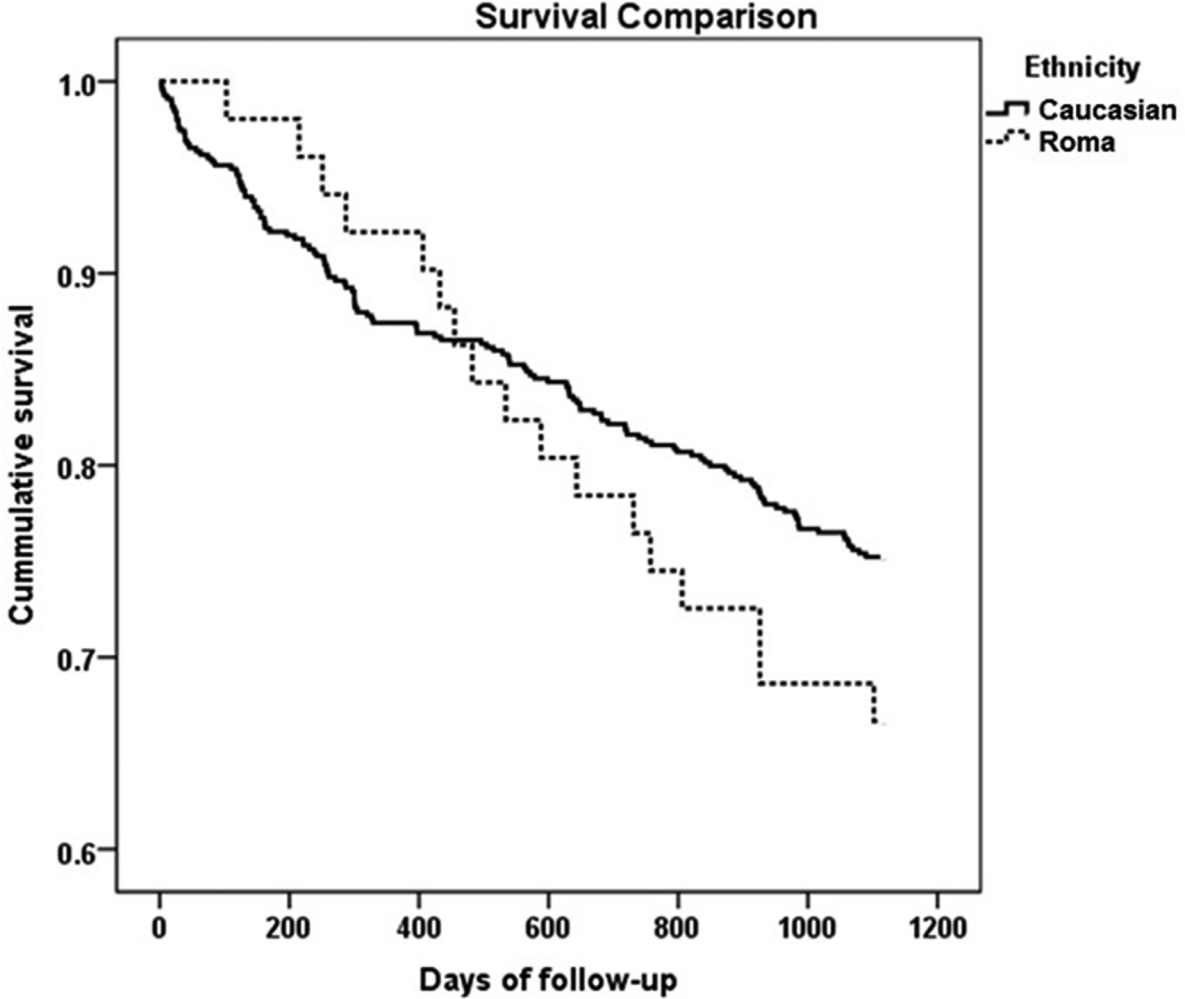
Survival analysis in dialysis patients: Roma vs. Caucasian.

An interesting observation is the crossing of the non-adjusted survival curves in the 424^th^ day of follow-up, with a more pronounced mortality in the Roma ethnic group after this moment. (Fig 1).

In order to assess the role of multiple factors in the hazard of mortality and, also, in order to analyze the mortality in an adjusted, standardized manner, we created a multivariate, stepwise, Wald backward, Cox-regression model, with the following variables considered for the backward algorithm: ethnicity (Roma vs. Caucasian), patient’s age, HD duration, BMI, hemoglobin, vitamin D levels, PO4, albumin, history of coronary artery disease, peripheral vascular disease, stroke, hepatic virus infections and type 2 diabetes. According to the backward Wald method and by defining an entry probability threshold of 0.05, respectively removal probability threshold at 0.10, the final equation was obtained after 7 removal steps. The final predictors accepted in the hazard equation were ethnicity, age, BMI, vitamin D levels, coronary artery disease and peripheral vascular disease. The excluded co-variates proved to be neither significant, nor marginally significant, having a p value for hazard ratio higher than 0.10.

According to our model Roma ethnicity (HR = 1.74; P = 0.035), higher age (HR = 1.03; P = 0.001) and the presence of coronary artery disease (HR = 1.67; P = 0.022) were associated with an increase in the risk of mortality in dialysis patients, while a higher value of vitamin D levels at baseline proved to have a significant protective impact (HR = 0.98; P = 0.017). In our model a higher body mass index also proved to have a protective action; however, it was only marginally significant (HR = 0.97; P = 0.068). The details of the multivariate Cox regression analysis are presented in Table 4.

**Table 4:** The results of multivariate Cox proportional hazards analysis.

Predictor	HR (95% CI)	p
Roma ethnicity	1.74 (1.04–2.91)	0.035
Age (per one year increase)	1.02 (1.01–1.04)	0.001
BMI (per one kg/m^2^ increase)	0.97 (0.93–1.01)	0.068
Cholecalciferol (per one ng/mL increase)	0.98 (0.97–0.99)	0.017
Coronary artery disease	1.67 (1.08–2.58)	0.022
Peripheral vascular disease	1.38 (0.96–1.97)	0.081

Our results suggest that after adjusting for the studied confounding factors, Roma ethnicity represents an important and significant risk factor for mortality in dialysis patients.

Since the survival curves were crossed at the middle of the studied period and thus the proportional hazards hypothesis may be biased [8, 9], we created two separate Cox models, one for the short time and another one for the long term survival (the second one, starting from the moment which the two curves are crossing– 424 days of follow-up).

According to these new models, the significant predictors for the short-term survival (424 days of follow-up) were age (HR = 1.021; p = 0.045) and peripheral vascular disease (HR = 1.686; p = 0.046) while BMI (HR = 0.952; p = 0.085) and the presence of coronary artery disease (HR = 1.903; p = 0.073) were only marginally significant (Table 5).

**Table 5:** The results of multivariate Cox proportional hazards analysis for the short-term survival (424 days).

Predictor	HR (95% CI)	p
Roma ethnicity	1.00 (0.40–2.52)	0.996
Age (per one year increase)	1.02 (1.00–1.04)	0.045
BMI (per one kg/m^2^ increase)	0.95 (0.90–1.01)	0.085
Cholecalciferol (per one ng/mL increase)	0.99 (0.97–1.01)	0.321
Coronary artery disease	1.91 (0.94–3.85)	0.073
Peripheral vascular disease	1.69 (1.01–2.82)	0.046

For the long-term survival, as defined the survival from day 425 to the end of the follow-up period (day 1125), we observed that Roma ethnicity (HR = 2.53; p = 0.004), age (HR = 1.02; p = 0.02) and cholecalciferol (HR = 0.98; p = 0.03) were significant predictors for mortality (Table 6).

**Table 6:** The results of multivariate Cox proportional hazards analysis for the long-term survival (425 to 1125 days).

Predictor	HR (95% CI)	p
Roma ethnicity	2.52 (1.34–4.76)	0.004
Age (per one year increase)	1.02 (1.01–1.04)	0.020
BMI (per one kg/m^2^ increase)	0.98 (0.94–1.03)	0.581
Cholecalciferol (per one ng/mL increase)	0.98 (0.96–0.99)	0.030
Coronary artery disease	1.47 (0.84–2.57)	0.178
Peripheral vascular disease	1.18 (0.71–1.96)	0.525

## Discussion

Data regarding the survival of Roma people with ESRD on maintenance HD are lacking. For the first time, we assessed the 3 year survival rate of Roma ethnicity from a large cohort of HD patients. Our data showed that the prevalence of Roma ethnics among HD patients is high. Roma peoples are initiating chronic HD at a younger age than Caucasians and are presenting more frequently higher phosphate levels. They are at a higher risk of dying at a younger age. Furthermore, in the long-term survival analysis, Roma ethnicity represents per se, a significant, independent risk factor for mortality in HD patients.

In our cohort the prevalence of Roma patients is high (8.5%), though, according to the 2011 Census, in our country, Roma ethnic minority represents only 3.3% of the population [10]. Similar findings have been reported by Kolvek et al. in one Slovakian ESRD cohort, the authors showing that the relative risk of ESRD for Roma was 1.34 times higher as compared to the majority population [5]. Also, epidemiological studies regarding racial/ethnic disparities in CKD revealed that the prevalence of ESRD is greater for minorities in the population of United States of America (USA) [11]. Thus, as compared to Caucasians, a 4-fold higher race-specific risk for developing ESRD was identified among African-Americans and a 1.5-fold higher risk in Hispanics [12].

According to our data, Roma patients with CKD initiated HD therapy at a significantly younger age compared to Caucasians, as the African-Americans in the USA [12]. Racial/ethnic minority groups are shown to have a more accelerated CKD progression [11, 13, 14] but the biological, physiological and socio-cultural differences which account for the faster decline of renal function remain unclear [15, 16, 17, 18].

The racial disparity in CKD could be related to the hereditary gene polymorphisms, to the variances in mineral bone disorder, nutritional status, dietary intake, inflammatory and oxidative status profile or in body composition [13]. Some psycho-social, cultural and economic particularities may also intervene: lack of health insurance and limited health care access, unemployment, cultural conflicts, poor communication abilities, poor education, high illiteracy level, social marginalization, poor living conditions, lack of ability to adapt to social rules, distrust in institutions or in the medical system. All these create a barrier leading to a late referral to a nephrologist, delayed diagnosis, low adherence to medical recommendations even if the access to the health care system is granted [15, 19, 20, 21, 22].

The data focusing on CKD in the Roma ethnicity are limited to few studies. Thus, scarce reports have shown a higher prevalence of primary renal diseases among Roma children, a higher frequency of nephropathies with a lower eGFR in Roma women, and a higher relative risk of ESRD (RR = 1.34) for Roma people as compared to Caucasian counterparts [23, 24, 5]. The average age of Roma ethnicity at HD initiation or renal transplantation is significantly lower than in the Caucasians [5, 25]. Our results are consistent with those presented above: high prevalence in the HD treated ESRD population and lower median age at the initiation of HD therapy in the Roma ethnic group.

The higher risk of CKD in Roma minority and the faster progression to ESRD seem to be associated with the similar disparities reported in African-Americans and/or Hispanics, but with some particularities. Giving the Indian origin of this population, specific genetic factors could be associated with the higher risk of CKD [26]. Recently, this minority has changed its traditional lifestyle, distinguished by a high level of physical activity and low energy diet to a more inactive lifestyle with an excess of caloric intake [2]. Thus, Roma people are at risk of developing obesity, insulin resistance, albuminuria, diabetes mellitus and cardiovascular disease [2]. Higher prevalence of unhealthy behaviors such as smoking, alcohol consumption has also been reported in these people [27]. Lower socio-economic conditions, marginalization, insufficient integration may be important contributors [4, 5, 22, 24, 27, 28]. Several studies have shown that chronic diseases in Roma people occur 5–20 times more frequently than in the general population and are associated with social exclusion, unfavorable housing conditions, inadequate nutrition and low education level [1, 29, 30].

In the last decade racial differences have been described in the regulation of bone and mineral metabolism in patients with ESRD [31, 32, 33, 34]. African-American HD patients present higher levels of plasma PTH, serum phosphorus and alkaline phosphatase as compared to Caucasians, and are more likely to exhibit high bone turnover abnormalities [31, 32, 34]. These findings have been attributed to a higher skeletal resistance to PTH or enhanced activation of a calcium-sensing receptor in the parathyroid gland [35]. In our cohort serum phosphorus and total alkaline phosphatase levels were significantly higher in Roma people than in Caucasians, though the dialysis efficiency (assessed as eKt/V) and treatment prescriptions with phosphate-binders, paricalcitol or active vitamin D were similar. The results are consistent with those described in the US African-Americans even if the genetic background is totally different, so, maybe other factors could be involved in our findings, for example lower adherence to diet recommendations. Roma patients presented increased serum levels of both albumin and phosphate as compared with their controls in this cohort, suggesting a diet with a higher protein content [36, 37, 38]. In HD patients with albumin levels above 3.5g/dl, the increase of phosphate concentration is associated with an increased risk of mortality [38, 39].

According to the results of the Global Burden of Disease 2013 study, CKD is a non-communicable cause of premature death, with a 90% increase over the last years [40]. The life expectancy of patients with ESRD treated with dialysis continues to be lower than for many cancer patients [12, 41]. Thus, a 5-year adjusted survival rate for all RRT patients in Europe was 59.7% and for the USA less than 35% [15, 41]. African-Americans and Hispanics in the USA seem to have a 13% to 45% survival-advantage under dialysis therapy compared to Caucasians [19]. Related to these findings, a survival paradox was described in these ethnic groups highlighting the survival advantage of HD therapy in contrast to the higher mortality rates in the general population [19, 42, 43, 44]. Various explanations have been hypothesized: genetic polymorphism, increased rates of major risk factors such as arterial hypertension and diabetes mellitus, racial differences in nutritional status, inflammation or mineral bone disease, differential sensitivity to dialysis, disparities in socioeconomic status affecting access to care or differences in mental coping mechanisms [20, 31, 45, 46].

The survival paradox does not apply to the Roma minority. Data regarding the mortality of Roma patients treated with HD are scarce and we are not aware of any other similar research. In our cohort of HD patients, Roma ethnicity represents an independent risk factor for mortality in long term survival analysis (HR = 2.52, P = 0.004). Moreover, Roma dialysis patients are dying at a younger age compared to Caucasians. In our study, the survival analysis revealed an interesting fact: Roma people presented a similar survival rate in the first 424 days of the study period, and a higher mortality rate after this date, as compared to Caucasian patients. It is possible that, at the initiation of HD therapy, Roma patients, being younger and healthier, had an initial better survival. Later on, they developed more severe complications at a higher rate which increased their mortality.

In the Roma minority from the general population or with kidney transplants the results seem to be similar. Jackson et al reported a life expectancy gap of 5–15 years in Roma people compared with the general population [47]. Furthermore, in a Hungarian adult kidney transplantation cohort of 1063 transplanted patients, 5.64% were Roma subjects and Roma ethnicity was independently associated with a 77% higher risk (HR = 1.77) of all-cause mortality [25].

The lower socio-economic status with subsequent limited access to health care system, lower adherence to medical treatment prescriptions and specific genetic background related to their North-Indian origin could be considered potential causes of the poorer outcome of the Roma population [2, 26, 48].

In Romania some of those explanations are not valid. Our country has a long-standing universal access to health care with a single, state managed financing system, generating a higher degree of homogeneity of quality of care and higher equity. Even under these conditions, the Roma population continues to experience a higher mortality rate.

Our study has several strengths: the large size of the studied cohort, recruited from one of the East European countries where Roma people represent the second-largest ethnic minority; a follow-up period of 3 years and the fact that it is the first study investigating Roma patients survival on HD therapy.

In our opinion the study has some limitations also. Firstly, we analyzed the risk of mortality by performing an extensive multivariate Cox regression analysis, but, as with all observational studies, we cannot exclude the possibility of residual confounding factors. Secondly, the severity of comorbidities could not be assessed. Finally some variables, such as education, income or hospitalization rate were not available in the patient’s medical records though they could have influenced the outcome.

## Conclusions

In conclusion, this study demonstrates that Roma people experience a higher prevalence of ESRD requiring dialysis. In the Roma minority group, patients initiate HD at a younger age, suggesting that CKD progression rate in the pre-dialysis period is higher in this ethnicity. No survival paradox could be evidenced in the Roma minority. According to our study, Roma patients with CKD treated with HD are at an increased risk of death and are dying at a younger age compared to their Caucasians counterparts. Further research is required to establish the causes of these findings.
